# Autofluorescence profiles define depot-specific adipocyte phenotypes in perivascular and non-perivascular adipose tissue

**DOI:** 10.1080/21623945.2026.2718625

**Published:** 2026-08-18

**Authors:** C. Javier Rendon, Allison D. Redondo-Aguilar, Daniel Vocelle, Matthew P. Bernard, D. Adam Lauver, Stephanie W. Watts, G. Andres Contreras

**Affiliations:** a Department of Large Animal Clinical Sciences, Michigan State University, East Lansing, MI, USA; b Department of Pharmacology and Toxicology, Michigan State University, East Lansing, MI, USA

**Keywords:** Adipocytes, PVAT, autofluorescence, adipose, flow cytometry

## Abstract

Perivascular adipose tissue (PVAT) is recognized as the functional fourth layer of the vasculature. Unlike non-PVAT depots, PVAT is continuously exposed to haemodynamic forces, potentially altering the morphometry of its adipocytes. While transcriptomics studies have identified diverse adipocyte populations, characterizing these cells remains difficult because their inherent fragility limits standard antibody-based phenotyping. Autofluorescence has emerged as a label-free alternative for characterizing cellular heterogeneity. However, autofluorescence-based characterization of PVAT adipocytes remains unexplored, and it is unknown if these cells exhibit distinct profiles reflecting their specific anatomical location. This study aimed to define the autofluorescence profiles of PVAT and non-PVAT adipocytes. Using Sprague-Dawley rats (*n* = 6), we assessed intrinsic fluorescence in adipocytes from PVATs of thoracic and abdominal aorta, and from mesenteric arteries alongside phenotype-matched non-PVAT counterparts: interscapular brown, subcutaneous, and retroperitoneal tissues. Using optimized methods to maintain adipocyte integrity, we demonstrate that thoracic and intrascapular exhibit similar autofluorescence profiles. In contrast, PVAT sites mesenteric and abdominal diverge across the yellow, red, and violet channels compared with non-PVAT depots, retroperitoneal and subcutaneous. In conclusion, PVAT and non-PVAT adipocytes exhibit depot-specific spectral patterns, with higher autofluorescence observed in PVAT cells, validating autofluorescence as a marker-independent approach for identifying adipocyte differences.

## New & noteworthy

Adipocytes are often viewed as functionally similar regardless of anatomical location, yet our findings demonstrate that they possess depot-specific autofluorescence signatures. By showing that PVAT adipocytes exhibit higher fluorescence intensity in specific emission ranges, we identify an overlooked optical property that may reflect underlying metabolic and structural differences. This provides a simple, label-free approach to distinguish adipocyte populations, offering new insights into depot-specific biology.

## Introduction

Perivascular adipose tissue (PVAT), the tunica adiposa of the vasculature, is a metabolically active layer that modulates vessel function through its secretome and the structural support that it provides to the vasculature [1]. Unlike non-PVAT depots, the tunica adiposa is continuously exposed to mechanical forces [2]. Research in the past three decades demonstrates that PVAT dysfunction contributes to the development of hypertension, atherosclerosis, and other cardiovascular diseases [3]. However, the PVAT adipocyte, the cell population that defines the tissue’s phenotype, is poorly characterized.

Phenotypically, adipocytes can be classified into three distinct types: white, brown, and brite/beige [4]. White adipocytes contain large, unilocular lipid droplets optimized for energy storage. Brown adipocytes possess multilocular droplets and dense mitochondrial networks that support thermogenesis via uncoupling protein 1 (UCP-1), a mitochondrial protein and hallmark marker of brown adipocyte function. Brite (or beige) adipocytes exhibit a hybrid morphology bridging the white and brown phenotypes and have the potential to become thermogenic (UCP1-expressing) under adrenergic stimulation. Anatomically, white adipocytes are the most common phenotype in visceral adipose tissue (AT) depots, including the omental, retroperitoneal, and perigonadal compartments. Brown adipocytes are found in the interscapular brown AT of rodents and, in humans, within the supraclavicular area and thoracic adipose depots, such as the PVAT surrounding the thoracic aorta [5]. Furthermore, some visceral (especially perirenal) and subcutaneous (especially inguinal AT) compartments contain a mixture of white and brite adipocytes. Because PVAT depots may also contain a heterogeneous mixture of these adipocyte phenotypes, current approaches to adipocyte population characterization require further refinement.

The most common approaches to phenotypic characterization of adipocytes include transcriptomic profiling, *in vitro* functional assays, antibody-based fluorescence microscopy, and flow cytometry. These methods typically rely on external dyes or fluorescent probes to label specific tissue components, enabling visualization of structures, cells, or proteins of interest. Recently, autofluorescence detection by flow cytometry has emerged as a marker-independent method to distinguish cell populations with different metabolic profiles in complex tissues [6,7].

Autofluorescence refers to the natural emission of light by biological structures upon excitation at specific wavelengths [8]. This phenomenon has been utilized in research and diagnostics to characterize cell populations in contexts such as cancer [9], ischaemia [10], and cell senescence [11]. Tissues exhibit multiple endogenous sources of autofluorescence, primarily driven by metabolic cofactors like nicotinamide adenine dinucleotide (NADH) and flavin adenine dinucleotide (FAD) [12,13]. Other significant contributors include extracellular matrix components such as collagen and elastin, as well as lipofuscin, a lipid-derived pigment associated with oxidative stress and lysosomal activity [11,14]. In flow cytometry, a major advantage of autofluorescence is the label-free, single-cell analysis it enables, avoiding artefacts from external dyes. By leveraging a broad range of excitation wavelengths and emission filters (multispectral autofluorescence) researchers can resolve distinct biochemical ‘fingerprints’ based on the unique spectral signatures of these endogenous fluorophores [15]. This approach enables rapid assessment of distinct phenotypic profiles without complex staining protocols, preserving cell viability for downstream applications. Currently, the use of autofluorescence to characterize mature adipocytes from PVAT and some non-PVAT depots remains unexplored. In this study, we compare the autofluorescence profiles of mature adipocytes from different PVAT depots and contrast them with those from non-PVAT sites with matching adipocyte phenotypes.

## Materials and methods

### Animals

This study used male Sprague–Dawley rats to limit sex-related variability and establish feasibility (*n* = 6; 8–10 weeks old; 300–350 grams of body weight; Charles River Laboratories, Portage, MI, USA, RRID: SCR_003792). Animals were kept under a 12:12 h light–dark cycle in a temperature-controlled room (22 °C) with environmental enrichment in standard cages. Rats had *ad libitum* access to standard chow (Research Diets, Cat N°D12450) and distilled water. All procedures were conducted in accordance with the ARRIVE guidelines for the reporting of *in vivo* experiments and approved by the Michigan State University Institutional Animal Care and Use Committee (protocol no. PROTO202400255) and complied with the Guide for the Care and Use of Laboratory Animals, 8th edition. For tissue collections, rats were euthanized (intraperitoneal injection of 60–80 mg/kg of pentobarbital), deep anaesthesia was verified by the lack of paw pinch and eye-blink reflexes, and death was assured by pneumothorax.

### Tissue collection

Thoracic PVAT (atPVAT) and abdominal PVAT (abPVAT) were isolated from the descending thoracic and abdominal aorta. Mesenteric PVAT (mesPVAT) was collected from mesenteric arteries (<200 microns). Control tissues or non-PVAT counterparts were harvested as follows: brown adipose tissue (BAT) from the interscapular region, subcutaneous adipose tissue (SCAT) from the inguinal region, and retroperitoneal adipose tissue (rpAT) from the retroperitoneal space dorsal to the kidneys. After harvesting, tissues were submerged in 1 mL of prewarmed Krebs-Ringer-phosphate buffer (KRBB) containing NaCl 135 mM; KCl 5 mM; MgSO4 1 mM; KH2PO4 0.4 mM; Glucose 5.5 mM; HEPES 20 mM pH 7.4 (Teknova, Cat N° H1030) and supplemented with 100 units/mL of penicillin; 100 µg/mL of streptomycin, 0.25 µg/mL of Amphotericin B and 50 µg/mL of Gentamicin following procedures previously described [16].

### Isolation of adipocytes

Adipose tissue samples were maintained at 37°C throughout the entire isolation process. Total of 150 mg of tissue were collected per site, samples were washed 3 times in KRBB, and three pieces of 50 mg each were cutted and transferred to a 1.5 mL tube. Tissues were minced while submerged in 1 mL of digestion solution (0.05 mg/mL Liberase^TM^ TL [Roche Diagnostics, Cat No. 5,401,020,001] dissolved in Hanks’ Balanced Salt Solution (HBSS) supplemented with 4% BSA [Fisher, Cat No. BP9706-100] and 10 mM HEPES), to create 1–3 mm tissue fragments. Samples were incubated at 37°C for 30 min in a rotisserie incubator to ensure continuous mixing of the solution. The digested material was filtered through a 150 µm cell strainer (Sigma Cat N° S4020-5AE) to remove undigested tissue fragments, then transferred to a new tube containing 1 mL of HBSS +4%BSA to dissolve any traces of Liberase^TM^. Samples from the same site were combined and then centrifuged at 100 *g*, RT for 5 min to separate the buoyant cells (adipocytes) from the stromal vascular fraction (SVF). To wash the adipocyte‑containing top layer, 1 mL of supernatant was gently aspirated using wide‑bore micropipette tips to allow aspiration of large adipocytes without affecting their viability. The collected supernatant was transferred into a new tube containing 1 mL of FACS buffer (1X PBS, 0.1% sodium azide [Fisher Scientific S227I‑25], 1% FBS [Gibco A5256801], and 1 mM EDTA [Lonza BMA51201]). Samples were centrifuged at 100 g at RT for 5 minutes. This wash step was repeated twice. Finally, the top layer of buoyant adipocytes was transferred to a new tube containing 250 µL of FACS buffer and was maintained in a water bath at 37°C for less than 30 mins for further analysis.

### Viability analysis

Viability analysis was performed in adipocytes after incubation with ViaStain^TM^ acridine orange (AO)-propidium iodine (PI) (Revvity CS2-0106-5 mL) for 10 mins at 37°C. Samples were analysed in the Cellometer Spectrum^TM^ Image Cytometry System (Revvity, Waltham). Images in triplicate were obtained using Spectrum v.5 software and analysed in ImageJ, and viability efficiency was determined as the percentage of AO counts/PI counts ×100.

### Flow cytometry data acquisition

Adipocyte samples were analysed using the Attune CytPix Flow Cytometer (ThermoFisher). Throughout the study, samples were acquired at an acquisition speed of 12.5 µL/min and Attune^TM^ Focusing Fluid was used as sheath fluid. (The Attune cytometer does not use nozzle). Adipocyte samples and collection tubes were maintained and analysed at 37°C throughout all experiments, as adipocyte lipids stiffen at lower temperatures, leading to increased cell breakage and clumping. To keep cells in suspension, samples were gently mixed by flicking the tube before acquisition (vortexing was avoided, as it can break adipocyte membranes). Gains were defined as follows for white and brite/beige adipocytes FSC:5, SSC: 225, and brown adipocytes FSC:100, SSC:275. Prior to acquisition, we examine cells through images and imaging parameters (area, perimeter, pseudo diameter, etc) in order to identify all cells that were morphologically similar, then we back gated them on forward scatter (FSC-A) versus side scatter (SSC-A) dot plot to exclude debris and dead cells based on size and granularity, and a gate was drawn around cell populations (intermediate-high FSC profile). Doublets and aggregates were excluded by plotting FSC-height (FSC-H) versus FSC-area (FSC-A); single cells fall along a linear diagonal (proportional area-to-height ratio), while doublets and larger aggregates deviate from this line due to increased area relative to height. A gate was drawn around the singlet population along this diagonal for further analysis. This step was repeated using SSC-H versus SSC-A as a secondary confirmation. Particle count and examination of images of individual adipocytes were used to confirm proper gating strategy. To quantify the area of each individual adipocyte event in the *adipo* gate, after gating out the doublets, the statistics average area was obtained and plotted as mean using rat as experimental unit. Autofluorescence measures were obtained by the median fluorescence intensity of each fluorescent channel after the following gating strategy: Adipo gate/ singlets gate. All data analysis was performed in FlowJo v10 software (Waters^TM^ BioSciences). The flow cytometry panel does not include a viability dye, which could indicate that dead cells or debris can be included in the autofluorescence analysis and may affect the interpretation of SSC properties or even autofluorescence across different channels. The autofluorescence profile measured as the median intensity were obtained from an average of (300–2000) adipocytes per depot per rat and were used as primary outcome for this study.

### Optical configuration and channel nomenclature

Autofluorescence data were acquired using the Attune CytPix. Table 1 details the optical configuration, including excitation lasers, channel nomenclature, and bandpass filters.

**Table 1.** Attune CytPix Laser and filter specifications.

Laser (nm)	Channel	Filter (nm)	Range (nm)
488	**BL1**	530/30	515–545
	**BL2**	695/40	675–715
561	**YL1**	585/16	577–593
	**YL2**	620/15	612–628
	**YL3**	780/60	750–810
637	**RL1**	670/14	663–677
	**RL2**	720/30	705–735
	**RL3**	780/60	750–810
405	**VL1**	450/40	430–470
	**VL2**	525/50	500–550
	**VL3**	610/20	600–620
	**VL4**	660/20	650–670
	**VL5**	710/50	685–735
	**VL6**	780/60	750–810

### Statistical analysis

Data are presented as mean ± SEM. Statistical analyses were performed using JMP Pro (v18), with the individual rat defined as the experimental unit and included as a random effect in the model. Based on a power analysis (β = 0.2) using our preliminary data, the sample size was selected. Fluorescence intensity represents the average of at least 200 adipocytes per group. Normality was assessed via the D’Agostino-Pearson test. No data was excluded and groups were compared using one-way ANOVA; however, for non-normally distributed data, the Kruskal-Wallis test was applied. Statistical significance was defined as *p* ≤ 0.05. No cofounders were controlled in this study.

## Results

**Figure 1.**
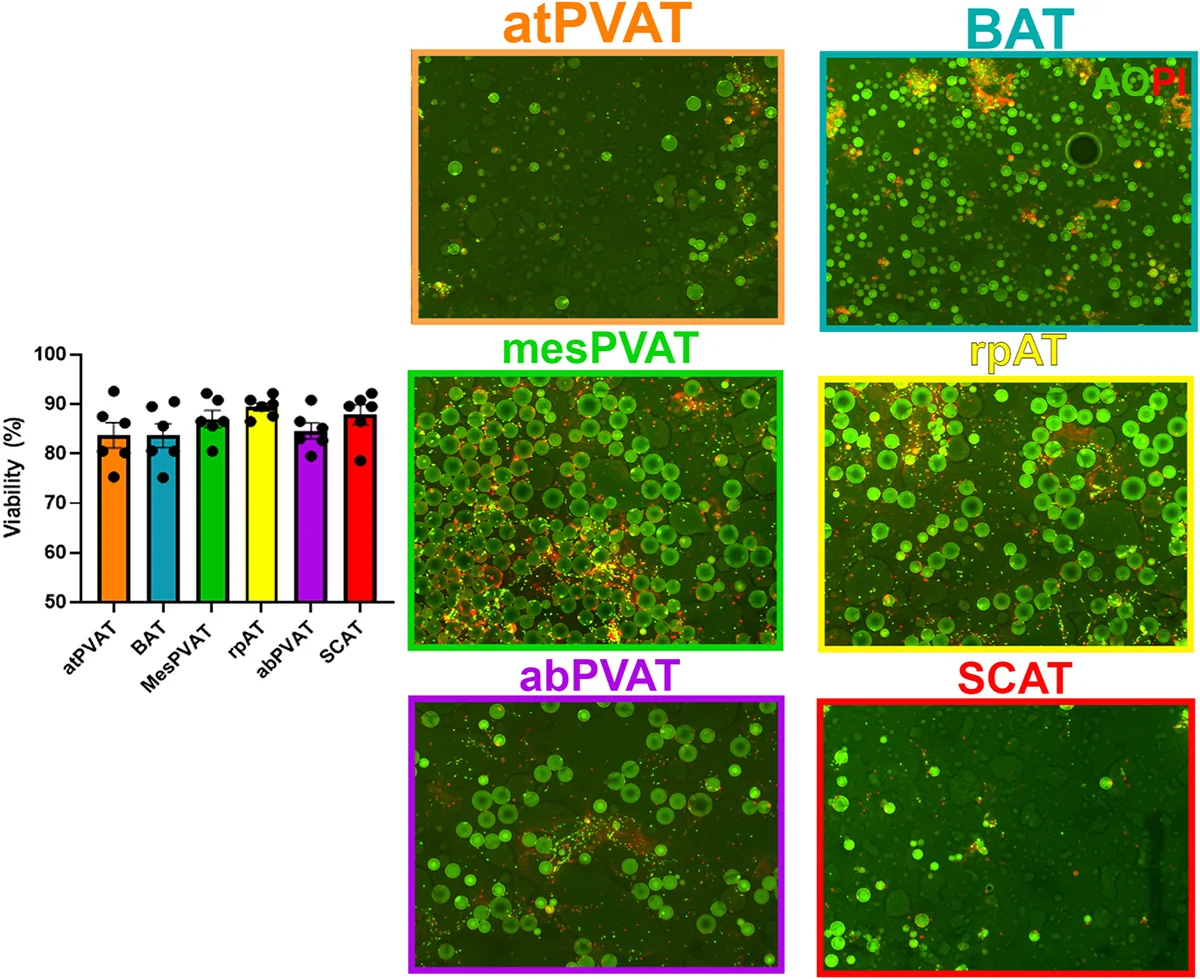
PVAT and non-PVAT adipocytes’ viability is maintained after enzymatic digestion. A) Quantitative assessment of adipocyte viability following enzymatic digestion. Viability is expressed as a percentage of live cells (acridine orange–AO^+^)/dead cells (propidium iodide–PI^+^) x 100 in different adipose depots: thoracic aortic PVAT (atPVAT), interscapular brown adipose tissue (BAT), mesenteric PVAT (mesPVAT), retroperitoneal adipose tissue (rpAT), abdominal PVAT (abPVAT), and subcutaneous adipose tissue (SCAT). Bars represent mean ± SEM. B) Representative Cellometer^TM^ Spectrum images showing live (AO^+^, green fluorescence) and dead (PI^+^, red fluorescence) cells from each adipose depot.

By implementing an adipocyte handling protocol to minimize mechanical and thermal stress, we obtained cell suspensions with high viability (>80%) and intact lipid droplets across all PVAT and non-PVAT sites (Figure 1). Specific optimizations included using wide-bore pipette tips, filtering samples through large-pore strainers (>150 µm), and maintaining cells at a constant 37°C. Additionally, we mixed the adipocyte suspensions immediately prior to cytometer acquisition to ensure sample homogeneity.

### PVAT and non-PVAT adipocytes exhibit distinct depot-specific flow cytometry profiles

**Figure 2.**
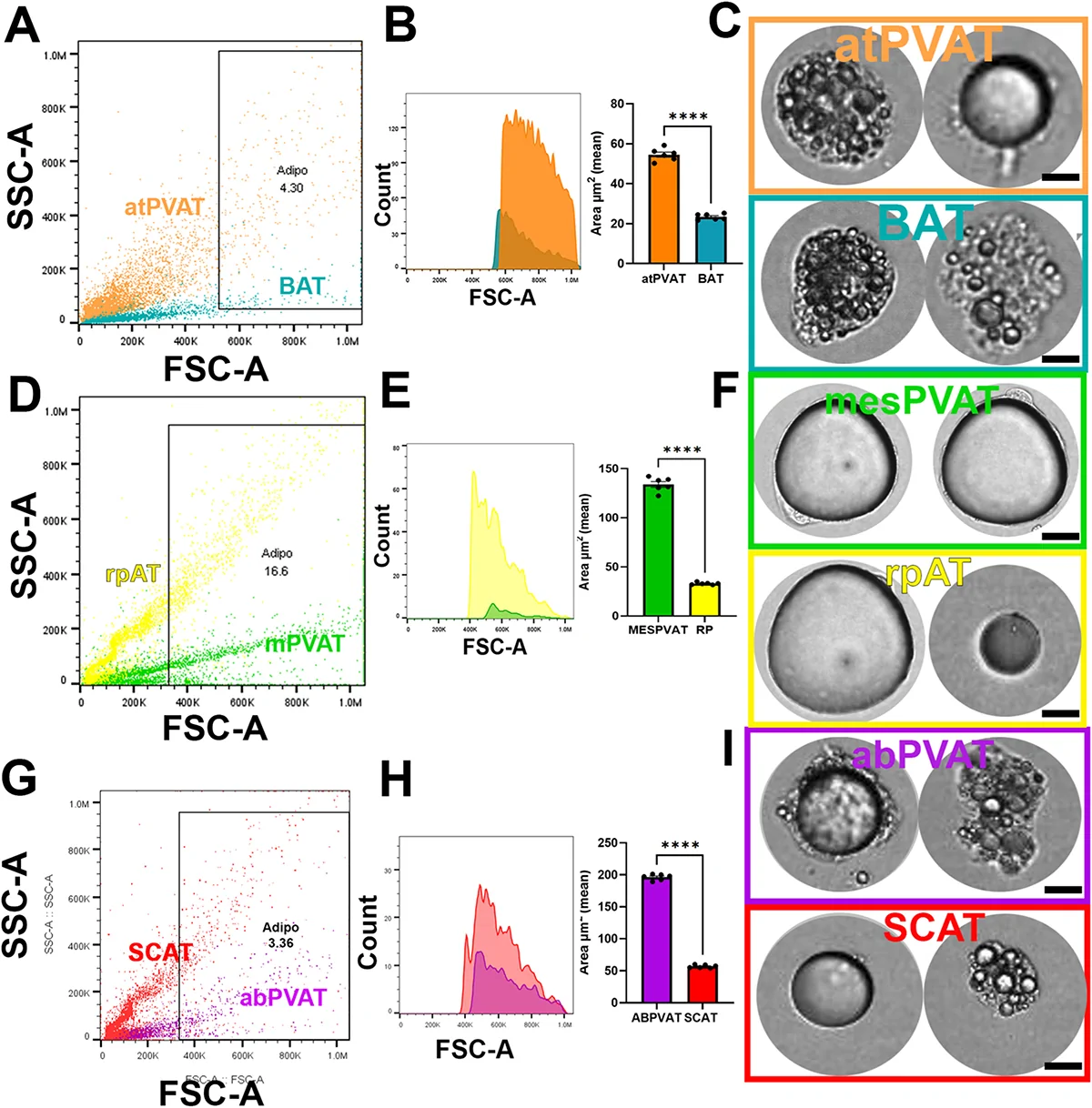
PVAT and non-PVAT depots differ in adipocyte size and SSC profiles. A) Representative scatter plots showing the distribution of forward scatter (*FSC*; x-axis) and side scatter (*SSC*; y-axis) for all events and gating for adipocytes of combined data from atPVAT (orange) and BAT (teal), D) combined data from rpAT (yellow), mesPVAT (lime), and G) combined data from SCAT (red) and abPVAT (green). B, E, H) Histogram of events from adipo gate showing distribution of counts across FSC signature between PVAT and non-PVAT depots (left) and bar graph summarizing cell area in µm2 in mean ±SEM (right). C, F, I) Representative images obtained via the Attune CytPix demonstrating the characteristic multilocular phenotype in PVAT depots and their counterparts, varying from unilocular to multilocular adipocytes. White adipocytes present nuclei in the periphery of the cell. Scale bar: 10 µm.

Scatter plot analysis of side scatter (*SSC*) vs. forward scatter (*FSC*) revealed distinct profiles for adipocytes from BAT and atPVAT, with clear separation between sites (Figure 2A). Adipocytes from atPVAT were larger than those from BAT (Figure 2B). Furthermore, Attune CytPix images demonstrated that atPVAT adipocytes contained larger lipid droplets and occasional unilocular cells; in contrast, BAT adipocytes were consistently multilocular (Figure 2C).

We observed a similar pattern of larger adipocyte size in PVAT sites within the visceral region. Adipocytes from mesPVAT displayed distinct scatter plot profiles compared to rpAT, with clear separation between the two sites (Figure 2D). This separation reflected the larger cell size (Figure 2E) and larger lipid droplets in mesPVAT vs. rpAT, as confirmed by Attune CytPix imaging (Figure 2F).

Finally, we compared abPVAT and SCAT, which contain a mixture of white unilocular and multilocular (brite) adipocytes. Scatter plot analysis showed clear separation between these depots (Figure 2G), with abPVAT adipocytes being larger than those from SCAT (Figure 2H). Attune CytPix images reflected these differences in cell diameter and lipid droplet morphology (Figure 2I).

### PVAT and non-PVAT adipocytes exhibit distinct depot-specific autofluorescence profiles

**Figure 3.**
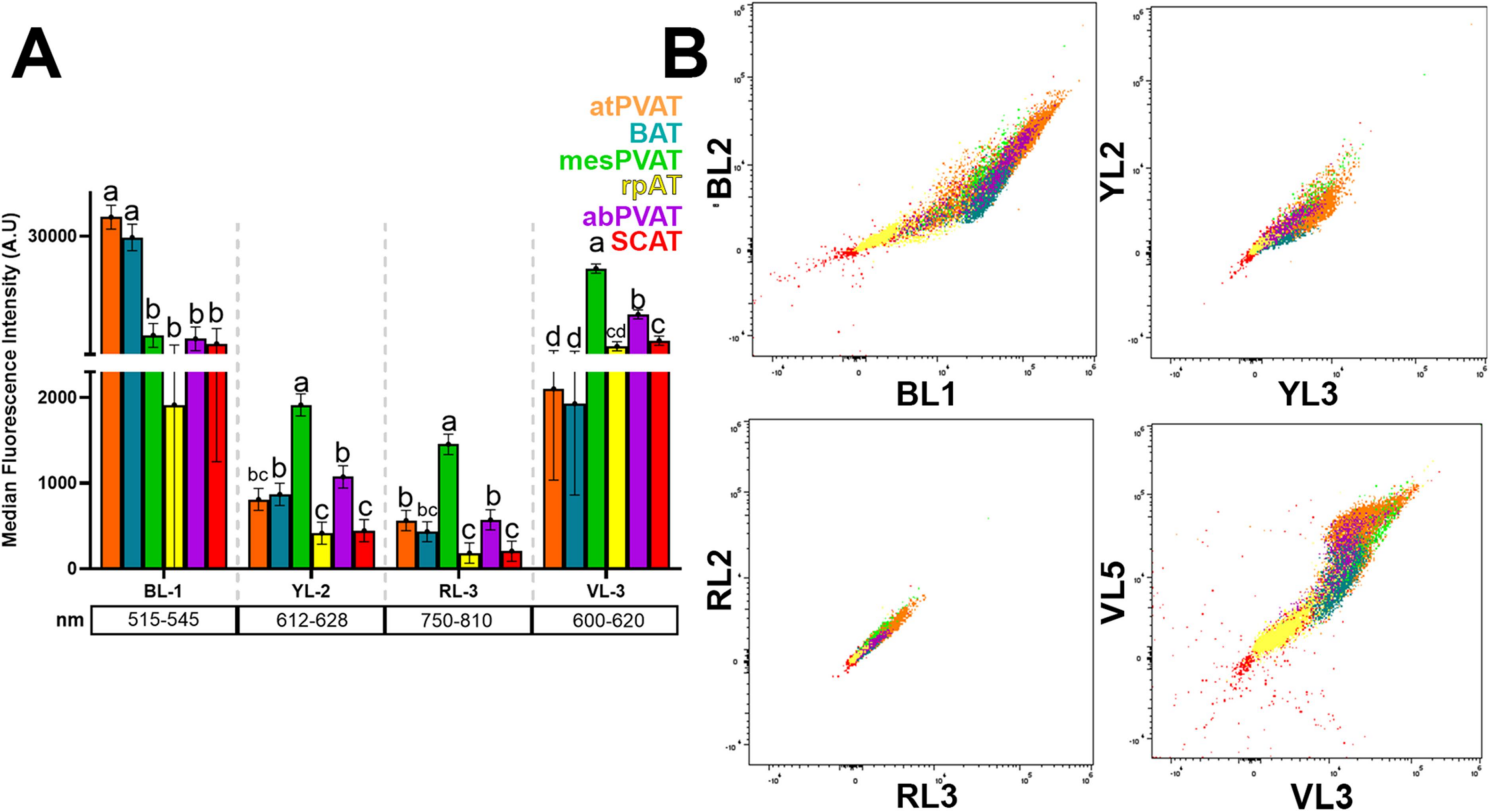
Autofluorescence profiles of PVAT depots and their non-PVAT counterparts. A) Quantitative comparison of median fluorescence intensity (*MFI*; y-axis) between PVAT and non-PVAT depots across blue (BL)-1, yellow (YL)-2, red (RL)-3, and violet (VL)-3 channels. Data are presented as median ± SE, different letters: *p* < 0.05. B) Scatter plots illustrating autofluorescence intensity in diverse filter channels across multiple blue (BL), yellow (YL), red (RL), violet (VL), and violet (VL) channels.

We analysed the autofluorescence of each adipose tissue depot across multiple spectral channels. Among the blue channels, we detected signal in BL2 (515–545 nm) across all sites. Depots with high brown adipocyte populations (i.e. atPVAT, BAT) exhibited higher BL2 autofluorescence than mesPVAT, rpAT, abPVAT, and SCAT (Figure 3A). We observed no differences in autofluorescence between atPVAT and BAT in this channel (Figure 3A).

Regarding the violet channels, VL3 (600–620 nm) yielded the highest signal. The VL3 autofluorescence pattern mirrored that of RL2, with the highest intensity in mesPVAT, followed by abPVAT, whereas the non-PVAT sites with large unilocular populations showed the lowest intensity (Figure 3A).

In the red spectrum, we detected emission in RL3 (750–810 nm) across all depots. RL3 autofluorescence was highest in mesPVAT, followed by abPVAT and the brown adipocyte predominant atPVAT and BAT, and was lowest in the unilocular-predominant non-PVAT sites, rpAT and SCAT (Figure 3A).

Among the yellow channels, YL2 (612–628 nm) was detected across all PVATs. PVAT depots with a higher abundance of unilocular adipocytes (mesPVAT, abPVAT) showed higher YL2 autofluorescence than those with multilocular adipocyte populations (BAT and atPVAT). Furthermore, mesPVAT and abPVAT exhibited higher YL2 intensity than their non-PVAT counterparts, rpAT and SCAT (Figure 3A).

## Discussion

Autofluorescence detection by flow cytometry provides a marker-independent method for characterizing cell populations. This study demonstrates that intrinsic autofluorescence offers a sensitive, label-free approach to distinguish adipocyte populations from PVAT and non-PVAT depots. Using optimized handling methods, we show that, despite morphological similarities, white adipocytes exhibit distinct autofluorescence profiles when comparing PVAT and non-PVAT depots. Differences in autofluorescence emission were not observed in adipocytes from BAT and atPVAT, which are predominantly brown. While the functional consequences of these differences remain unknown, exposure to haemodynamic forces may influence these autofluorescence profiles.

### Optimization of adipocyte handling for flow cytometry analysis

Preparing single-adipocyte suspensions for flow cytometry is difficult due to the physical properties of these cells. Adipocytes have a lipid content exceeding 90% of cellular volume [17], resulting in buoyancy, large size, and fragility. These characteristics increase their sensitivity to common cytometry hazards, including shear stress, pressure, narrow nozzle passage, and frequent pipetting [18,19]. Additionally, high lipid content renders these cells susceptible to the low temperatures (below 37°C) typically used in flow cytometry protocols [20]. Our optimized adipocyte handling protocol prioritizes maintaining cell integrity by using wide-bore pipette tips, filtering samples through large-pore strainers (>150 µm), maintaining samples at 37°C to preserve cell structure, and gently mixing samples during cytometer acquisition. These approaches are consistent with previous technical reports emphasizing that adipocytes are among the most fragile cell types handled in flow cytometry applications [18–20].

### Adipocyte flow cytometry profiles

Our findings align with previous reports evaluating brown adipocyte area in atPVAT [21] and BAT [22,23]. Although few studies have tried to compare adipocyte size between depots using microscopy imaging analyses [24,25], brown phenotypes remain a challenge to analyse with imaging, here using flow cytometry we found that BAT adipocytes are smaller than those in tPVAT. Several factors may explain these size differences. First, atPVAT and BAT have distinct ontogeny. BAT adipocytes are ‘classical’ brown adipocytes derived from Myf5^+^/Pax3^+^ progenitors in the paraxial mesoderm [26,27]. In contrast, tPVAT adipocytes have heterogeneous origins, including Myf5^−^, SM22α^+^, and Osr1^+^/mesoangioblast-like cells [27,28]. In addition to ontogenic differences, these depots exist in contrasting tissue environments. While BAT is located in the interscapular region, atPVAT surrounds the thoracic aorta and is exposed to constant haemodynamic forces [2]. Although a direct cause-effect relationship between adipocyte phenotype and exposure to mechanical forces remains unestablished, this mechanical exposure may influence metabolic responses and, consequently, adipocyte size [29]. Yet we recognize that our analyses may exclude a subset of mature adipocytes that exceed the cytometer’s fluid sheet size, potentially introducing size-dependent bias into the results.

Our results indicate that mesPVAT had larger adipocytes than rpAT. This result contrasts with a report from Boque and colleagues reporting higher average adipocyte area and higher proportion of large adipocytes (90–130 µm) in rpAT (15%) vs. mesenteric AT (3.2%) in Wistar rats [30]. In contrast, Hausman et al., reported no differences in size between mesenteric and rpAT [31]. However, it is important to note that many studies report the size of mesenteric AT and not the actual mesPVAT. Our methods harvest adipocytes only from mesenteric arteries that are up to 200 µm of diameter.

Depots with heterogeneous populations of adipocytes, including unilocular and multilocular (i.e. abPVAT and SCAT), have the capacity to expand their multilocular adipocyte populations during cold exposure [32]. Our results here demonstrate larger cells in abPVAT compared to SCAT. Although characterizations of the morphometry of abPVAT adipocytes are limited, they indicate an average size of 110–160 µm [22,33] that coincides with our findings. The SCAT adipocyte size reported in the present study also coincides with the 30–60 µm size reported in previous studies in rats [34,35].

#### PVAT and non-PVAT adipocytes autofluorescence profiles

We detected fluorescence in the BL1 (Ex: 488 nm; Em: 515–545 nm) channel across all evaluated AT sites, with the strongest emission in depots rich in multilocular adipocytes, such as tPVAT and BAT. The high intensity of the signal in this channel is likely due to the high mitochondrial density of these brown adipocytes; these organelles contain flavin adenine dinucleotide (FAD), which emits autofluorescence in the 505–550 nm range [36]. In contrast, unilocular adipocytes have a lower FAD content, likely explaining their reduced emission in the BL1 channel [37]. Previous studies have used BL1 emission to detect the emergence of thermogenic ‘beige’ adipocytes within white AT and to monitor thermogenic activity in brown adipose depots [37].

Fluorescence in the YL2 (Ex: 620 nm; Em: 612–628 nm) and VL3 (Ex: 405 nm; Em: 600–620 nm) channels was highest in mesPVAT and abPVAT. Although the exact source of these emission patterns remains unclear, the blood-derived metabolite hematoporphyrin [excited by both yellow-green (561 nm) and violet (405 nm) lasers] shows intense emission in the 600–630 nm range [38]. Given the immediate proximity of PVAT to the vascular wall, it is possible these cells accumulate higher concentrations of porphyrins and related haem-derivatives. Indeed, such accumulation has been demonstrated in the smooth muscle cells of large vessels under both normotensive and hypertensive states [39].

Similarly, autofluorescence in the RL3 channel (Ex: 633–640 nm; Em: 750–810 nm) was highest in mesPVAT, intermediate in tPVAT and abPVAT, and lowest in non-PVAT sites (rpAT and SCAT). The source of autofluorescence in this near-infrared (NIR) range is less characterized in adipose tissue. Given mesPVAT and abPVAT proximity to the intestinal lumen, a possible source for these autofluorescence patterns is the presence of dietary metabolites such as chlorophyll and its derivatives [40]. These products are potent emitters in the NIR range when excited by red lasers (633–640 nm) and may be absorbed by the mesenteric depot and the abPVAT [40].

Altogether, our results indicate that PVAT depots exhibit higher autofluorescence patterns than their non-PVAT counterparts. This increased fluorescence is likely attributable to both a higher concentration and a greater diversity of endogenous (e.g. FAD, porphyrins) or exogenous (e.g. chlorophyll) fluorophores. Since there are phenotypic similarities in thoracic PVAT adipocytes between rats and humans, we predict that these results may be translatable to human research [41,42]. Future studies integrating multimodal optical analysis, lipidomics, and spatial transcriptomics will help define the contributions of individual fluorophores and better characterize PVAT’s biology, advancing our understanding of their role in vascular dysfunction, obesity, and hypertension.

In the present study, flow cytometry-based sizing of adipocytes is inherently constrained by the Attune CytPix’s 150 µm diameter limit, which may not capture the full spectrum of adipocyte sizes present in vivo. Very large, lipid-laden adipocytes can exceed the upper limit of detection or be excluded during acquisition, potentially skewing our analysis towards smaller and intermediate-sized cells. Because adipocyte hypertrophy is itself associated with metabolic dysfunction, the underrepresentation of the largest cells could affect the interpretation of size distribution differences between groups. Future studies incorporating complementary sizing approaches would help validate and extend these findings across the full range of adipocyte sizes. The present study focused on developing and optimizing the methodology of analysing mature adipocytes, and therefore, to limit the confounding variables related to sex [43] only male rats were used in this study.

## Conclusion

Autofluorescence serves as a marker-independent method to characterize adipocyte heterogeneity across adipose depots. Depots enriched in multilocular adipocytes, such as BAT and tPVAT, show higher blue-channel autofluorescence consistent with mitochondrial content, whereas unilocular-dominant depots show lower signal in this range. Among morphologically similar unilocular-dominant depots, PVAT adipocytes display higher autofluorescence in yellow, red, and violet channels than non-PVAT counterparts. These spectral differences indicate that autofluorescence detects depot-specific variation not evident from morphology. These findings support autofluorescence profiling as a label-free approach to distinguish adipocyte populations and advance the understanding of PVAT biology.

## Data Availability

Derived data supporting the findings of this study are available on the following DOI: http://org/10.6084/m9.figshare.32374152.
